# Influence of Different Mouth Rinsing Agents on Friction During Sliding Mechanics Between Orthodontic Metal Brackets and Stainless Steel Archwire: A Comparative In Vitro Study

**DOI:** 10.7759/cureus.41224

**Published:** 2023-06-30

**Authors:** Datla Himabindu, Pappala Venkata Prasanna, Vazrala Vamsi Krishna Reddy, Shaik Farhatulla, RSVM Raghu Ram

**Affiliations:** 1 Orthodontics and Dentofacial Orthopaedics, GSL Dental College & Hospital, Rajahmundry, IND; 2 Orthodontics and Dentofacial Orthopaedics, GSL Dental College & Hopital, Rajahmundry, IND

**Keywords:** ozone-infused oil-pulling solution with coconut oil, charcoal mouthwash, sodium fluoride mouthwash, chlorhexidine gluconate mouthwash, artificial saliva, sliding mechanics, metal brackets, lubrication, frictional force

## Abstract

Aim and objectives

The aim of this in-vitro study is to evaluate and compare the effect of various mouth rinsing agents on frictional resistance through sliding mechanics among orthodontic metal brackets and stainless steel (SS) archwire.

Materials and methods

Each group comprised 15 samples of maxillary first premolar pre-adjusted edgewise SS movable, un-bonded brackets (Koden Inc., United States) which were welded with a power arm, where 100 g of the load was suspended. Brackets were ligated with elastic modules (Koden Inc., United States) onto the perspex sheet along with 0.019" x 0.025” SS archwire (Classic Orthodontics, United States) and were suspended from the upper component of the Instron machine. The coefficient of friction was tested in dry conditions (control), artificial saliva (AS) (Wet Mouth, ICPA Health Product Ltd., India), 0.2% chlorhexidine gluconate (CHG) mouthwash (Hexidine, ICPA Health Product Ltd., India), 0.05% sodium fluoride (NaF) mouthwash (ACT Anti-Cavity Fluoride Mouthwash, Sanofi Company, United States), charcoal (CC) mouthwash (Hello Activated Charcoal Extra Freshening Mouthwash, Hello Products LLC, United States) and ozone-infused oil-pulling solution with coconut oil (O_3_) (O_3_ Essentials, Health Ranger Store, United States). In order to calculate the coefficient of friction, 50 L was added* *to the test sample while moving at a 5 mm/min crosshead speed. The groups were compared using the one-way analysis of variance (ANOVA), and Tukey's post hoc analysis was performed for multiple pairwise comparisons.

Results

The coefficient of friction with the highest mean values was observed with the control group (2.01), followed by AS (1.79), and the least with O_3_ (1.15). Statistically significant differences were observed with almost all groups of mouth rinsing agents, but NaF is significant with CHG and CC. However, CHG did not have any significant difference from CC.

Conclusions

Lower coefficient of frictional values were observed with the ozone-infused oil-pulling solution with coconut oil during sliding mechanics between metal brackets and stainless steel archwire. Almost all the mouth rinsing agents showed a significantly different coefficient of friction value.

## Introduction

Friction influences tooth movement efficiency and anchorage control in orthodontics. Excessive friction impedes sliding, while moderate friction supports anchorage. However, relying solely on friction for anchorage can result in unintended movement. Orthodontists minimize friction to optimize treatment outcomes, ensuring applied forces surpass bracket-archwire friction for successful sliding tooth movement [1, 2]. A clinician's sound knowledge of the factors influencing friction, where static frictional force is generally higher than dynamic friction [3], was of utmost importance for the effective management and optimization of orthodontic treatment. In orthodontics, tooth movement occurs through frictionless or sliding mechanics. The frictional loss of applied force was around 60%, varying from 12% to over 70% in studies. The remaining force is transferred to supporting structures, requiring an adequate translational force within the optimal force and moment-to-force ratios for effective tooth movement [4].

Frictional resistance in orthodontics was influenced by physical factors like bracket and archwire properties, and ligation type, as well as biological factors such as the presence of saliva and oral flora [3]. Stainless steel (SS) brackets exhibited the lowest frictional forces, with certain wire combinations resulting in less than 100 g of friction. Sintered SS brackets had approximately 40-45% less friction than cast-formed SS brackets [5]. Among different bracket-archwire combinations, pre-adjusted edgewise bracket systems had the utmost friction. SS archwires had the least friction, and as wire diameter increased, the play among the bracket slot and wire decreased, resulting in higher friction with rectangular wires compared to round wires [5, 6]. Frictional resistance, predominantly influenced by the nature of ligation, exhibited greater static friction in the figure of eight modules compared to conventional modules [7,8].

Fixed orthodontic therapy creates additional plaque-retentive sites where microorganisms adhere and multiplied, forming a biofilm that increases friction [3]. To avoid plaque build-up, practitioners might have advised patients to use mouthwash. Due to the corrosive nature of brackets and archwires, the use of mouthwash during orthodontic treatment might have impaired the mechanical characteristics and surface quality of orthodontic wires, thereby affecting frictional resistance [9]. Chlorhexidine Gluconate (CHG) mouthwash demonstrated significantly higher friction due to the release of hydrofluoric acid by bactericidal effect [10, 11].

Oil pulling, a modified form of oil gargling, was used to prevent oral malodor, dental decay, and bleeding gums. Moreover, it was shown to decrease colony-forming units by 44.5% compared to the CHG group, providing significant oral health benefits in patients with fixed orthodontic appliances [12]. Oil's high lubricity, attributed to the oxygen atoms in ester molecules forming a monolayer on metal surfaces, may contribute to friction reduction [13]. Powdered charcoal, used in toothpaste for teeth whitening, has a mildly abrasive nature that could have helped remove surface stains but could also have caused enamel wear, resulting in a yellowish appearance [14]. No relevant evidence existed regarding the impact of activated charcoal mouthwash on friction.

Previous literature did not report on the impact of ozone-infused oils and activated charcoal mouth rinses on frictional resistance among orthodontic brackets and archwires. Hence, this study aims to compare the effects of ozone-infused oils and activated charcoal mouth rinses with other regularly used mouthwashes versus dry conditions, assessing their lubricating effect in terms of frictional characteristics.

## Materials and methods

Methodology

Ninety samples of perspex sheets were used in the current investigation based on preliminary data and power analysis obtained by the pilot study, which considered a confidence level of 95% and power of 80%. In each experimental condition, 15 samples were shown to be sufficient to satisfy the requirements (alpha = 0.05 and power = 80). The study was permitted by the Institutional Ethics Committee of the GSL Educational Institution, Rajamahendravaram, Rajahmundry, India (GSLDC/IEC/2021/006).

The present study takes the reference of Kapila et al. (1990) for standardising the in-vitro methodology for determining the frictional characteristics of an archwire during sliding mechanics [2]. A 10 x 2.5 cm perspex sheet of 6 mm thickness was used in the experiment. The sheet had a non-working area of 4 cm at the bottom and was fixed to the lower base compartment of the Instron universal testing device. Approximately 2.5 cm below the top of the sheet, a 1 x 1 cm cut was made in the center to suspend the movable bracket, to which a weight was added. The sheet was marked to indicate the placement of four maxillary first premolar pre-adjusted edgewise stainless-steel (SS) brackets (Koden Inc., United States) at regular intervals of 8 mm, representing the ideal inter-bracket distance. The brackets were lined up in a straight line, with two above and two below the initial cut surface. A 0.019” x 0.025” SS straight wire (Classic Orthodontics, United States) was used as a reference point.

**Figure 1:**
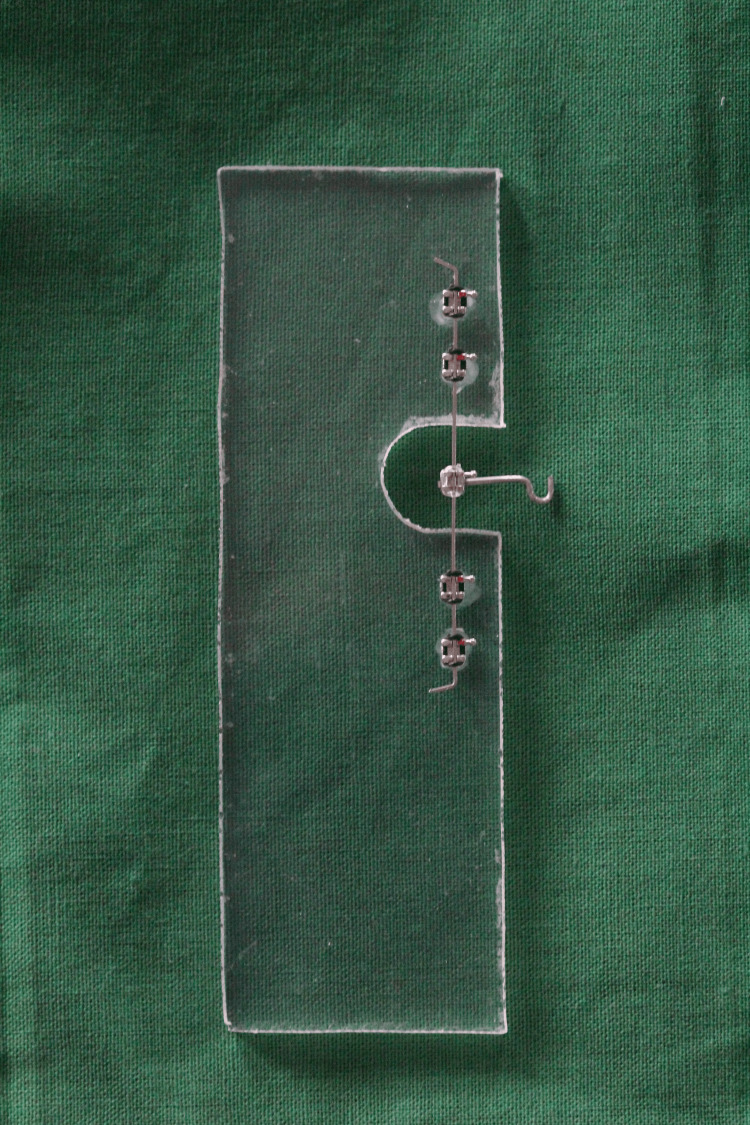
Prepared model of orthodontic sliding mechanics.

To prepare the sheet for bonding the brackets, the surface was roughened using a vulcanite bur. A light cure primer was applied using an applicator brush and cured for 20 seconds with a light-emitting diode curing unit. Light cure adhesive was then applied to the base of each bracket and cured for 40 seconds for bonding to the ventral surface of the sheet. A power arm with a hook was created using a 19-gauge SS wire. It was placed in contact with the base of the free movable bracket using dental wax, and spot welding was performed to secure it in place. Clear elastic modules were used to ligate the movable bracket and power arm to the 0.019" x 0.025" SS straight wire (Koden Inc., United States). Color-coded elastomeric modules were used to ligate all of the bonded brackets to the SS straight wire; different modules were used for each experimental group (Figure 1).

**Figure 2:**
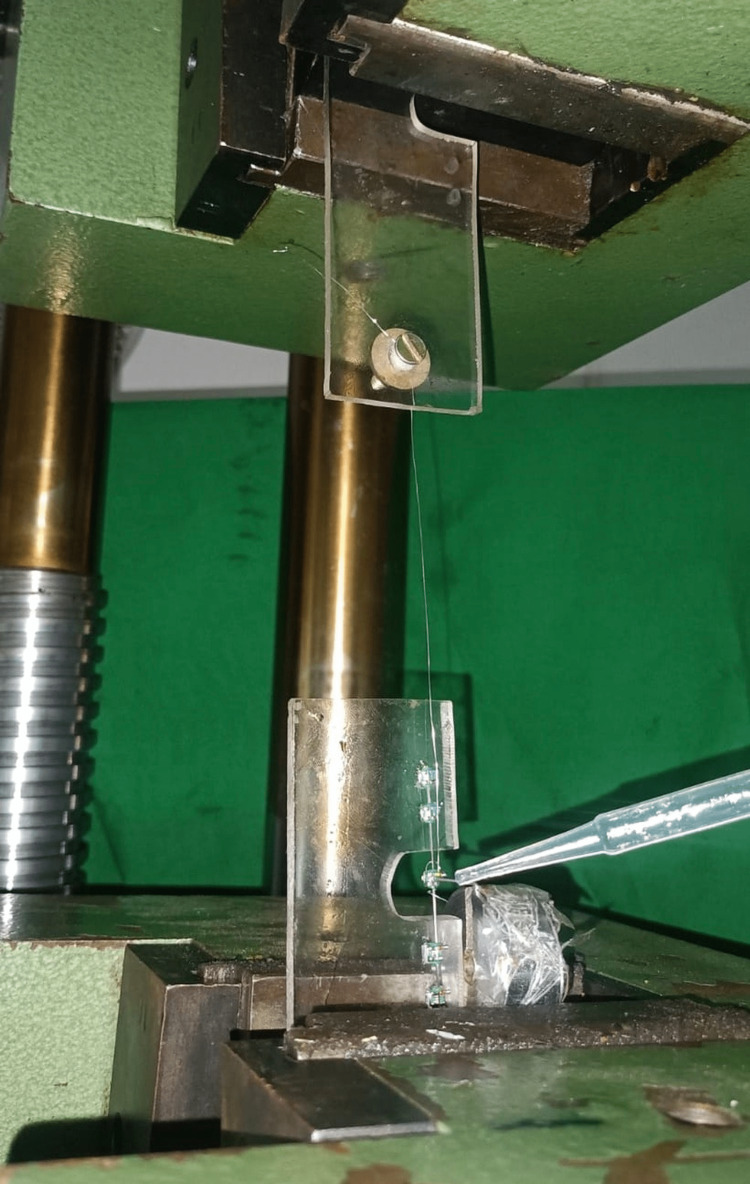
Application of mouth rinsing agents at the wire-bracket interface using a micropipette.

A weight of 100 g was held over from the power arm, and the sheet was ready to be placed in the universal testing device (Instron UTES-40-HGFL, Instron, Bengaluru, India)** **for further testing and analysis. Out of the 90 samples used in this study, 15 perspex sheets were divided for each group (a total of six groups) and labelled. Ninety samples were prepared on the perspex sheet where the free bracket was attached with the help of a clear elastic module in all the samples. These jig samples were distributed equally. The free movable bracket was suspended through 0.009” SS ligature wire from the upper component of the Instron, which is the load cell, while the perspex sheet was mounted on the lower component. Before the beginning of each test, a trial run was conducted without weight for the free sliding of wire in the bracket slot. A weight of 100 g was added to the power arm and 50 µL of the group-specific mouth rinsing agents were added to the wire-bracket interface using a micropipette to the movable bracket on the jig (Figure 2) [2, 15].

The groups include artificial saliva (AS) (Wet Mouth, ICPA Health Product Ltd., India); 0.2% chlorhexidine gluconate (CHG) mouthwash (Hexidine, ICPA Health Product Ltd., India); 0.05% sodium fluoride (NaF) mouthwash (ACT Anti-Cavity Fluoride Mouthwash, Sanofi Company, United States); charcoal (CC) mouthwash (Hello Activated Charcoal Extra Freshening Mouthwash, Hello Products LLC, United States), ozone-infused oil-pulling solution with coconut oil (O_^3^_) (O_3_ Essentials, Health Ranger Store, United States) and control group (dry condition). All of these sample groups were sent through Instron at 5 mm/min crosshead speed under a full-scale load of 100 N. The clinical retraction force was represented by the load-cell readout on the Instron machine, where frictional resistance makes up a portion of that force. The force needed for movement (p) and frictional force (F = p/2) were calculated to determine the variance between the load cell reading and the load that was added to the power arm. The coefficient of friction was calculated using this frictional force: f = f/N (where N is the load imparted to the power arm).

Final calculations, records, and statistical analysis were performed on all the resulting numbers. The software SPSS version 20 (IBM Corp., Armonk, United States) was used to analyse the data. The Kolmogorov-Smirnov test and descriptive statistics were used to evaluate the normality of the study's data. The data were analysed using ANOVA to compare the outcome parameters among the six study groups. Tukey's post hoc testing was performed for multiple pairwise comparisons of the coefficient of friction.

## Results

Intergroup comparisons of the coefficient of friction revealed that the highest mean values for µ were observed in the dry condition (2.01), followed by AS (1.79). The least mean values for the coefficient of friction were observed with O_3_ (1.15). Using ANOVA, the differences in the coefficient of friction between the groups were found to be statistically significant (p<0.001) (Table 1).

**Table 1:** Comparison of coefficient of friction between the study groups. ANOVA; p ≤ 0.05 considered statistically significant; * denotes statistical significance

Group	n	Mean	Standard Deviation	P-value	
					Dry condition	15	2.0173	.27650	<0.001*	
Artificial saliva	15	1.7907	.18507						
0.2% Chlorhexidine gluconate mouthwash	15	1.5260	.21695						
Charcoal mouthwash	15	1.4507	.15059						
0.05% Sodium fluoride mouthwash	15	1.6093	.16977						
Ozone-infused oil-pulling solution with coconut oil	15	1.1573	.10450						

The coefficient of friction between the different study groups was compared using Tukey's post hoc tests. Statistically significant differences were observed in multiple pair-wise comparisons. The dry condition showed significantly higher friction compared to AS (mean difference = 0.22667, p = 0.021), CHG (mean difference = 0.49133, p < 0.001), CC (mean difference = 0.56667, p < 0.001), NaF (mean difference = 0.40800, p < 0.001), and O_3_ (mean difference = 0.86000, p < 0.001). Among the other comparisons, significant differences were found between AS and CHG; CC and O_3_. No discernible differences were found between AS and NaF. The comparisons between CHG and CC; CHG and NaF; and CC and NaF did not yield statistically significant differences. However, significant differences were found between CC and O_3_; NaF and O_3_; and NaF and O_3_. These findings highlight the varying levels of friction coefficients among different study groups (Table 2).

**Table 2:** Multiple pair-wise comparisons for the coefficient of friction between the study groups. Tukey’s post hoc tests; * denotes statistical significance with p ≤ 0.05

Reference Group (X)	Comparison Group (Y)	Mean Difference (X-Y)	Significance	
				Dry condition	Artificial saliva	.22667	.021*	
Chlorhexidine gluconate mouthwash	.49133	.000*		
Charcoal mouthwash	.56667	.000*		
Sodium fluoride mouthwash	.40800	.000*		
Ozone-infused oil-pulling solution with coconut oil	.86000	.000*		
Artificial saliva	Chlorhexidine gluconate mouthwash	.26467	.004*	
	Charcoal mouthwash	.34000	.000*	
	Sodium fluoride mouthwash	.18133	.111	
	Ozone-infused oil-pulling solution with coconut oil	.63333	.000*	
Chlorhexidine gluconate mouthwash	Charcoal mouthwash	.07533	.889	
	Sodium fluoride mouthwash	-.08333	.840	
	Ozone-infused oil-pulling solution with coconut oil	.36867	.000*	
Charcoal mouthwash	Fluoride solution	-.15867	.219	
	Ozone-infused oil-pulling solution with coconut oil	.29333	.001*	
Sodium fluoride mouthwash	Ozone-infused oil-pulling solution with coconut oil	.45200	.000*	

## Discussion

Under dry conditions, a study by Leal et al. (2014) explained that friction was amplified as the surface contact increased [15]. Another hypothesis offered by Almeida et al. (2019), was that the wire and bracket asperities had intimate touch with each other, likely causing little debris to develop and enhancing friction [16]. In the present study, the coefficient of friction of the SS bracket against SS wires is 2.017. Our recorded values supported Almeida et al. (2019), where frictional resistance was observed as 2.14 [16]. These results also substantiate the results of Leal et al. (2014) with 2.23 and with less resistance to friction [15]. Studies done by Baker et al. (1987) show that artificial saliva reduced the frictional force value due to its viscosity, which is at 14 centipoises [17]. But the more recent study by Almeida et al. (2019) shows AS generated more friction than human saliva, distilled water, and dry conditions. This was explained as AS had the same rheological properties that limit its adsorption capacity and film formation [16].

Stannard et al. (1986) stated that dry condition was found to decrease the coefficient of friction irrespective of the alloy wires compared to lubricant. In general, lubrication lowers friction, but the liquid must be a lubricant to do so. It is well known that water and polar liquids increase adhesion, or the attraction between polar materials, which raises friction [18]. The present study supports Baker et al. (1987) where the coefficient of friction is recorded as 1.79 [17]. But, on the contrary, Almeida et al. (2019) reported higher frictional values in the range of 2.21 [16]. Studies supporting the CHG mouth rinse done by Emadian et al. (2021) observed a high rate of friction for the CHG group [9]. However, Omidkhoda et al. (2017) show that SS wires with GHG had the highest pitting surface, which in turn increased the friction when compared to Nickel Titanium wires [19]. Additionally, Tanti et al.'s (2018) research test results revealed that CHG had the maximum release of nickel ions from the SS brackets, where corrosion and surface roughness had a direct relationship [20].

However, research by Hosseinzadeh et al. (2013) did not reveal any appreciable differences in the frictional resistance between the SS brackets and the orthodontic archwires or the surface roughness of archwires [21]. The current study showed results that frictional resistance with CHG was within the range of 1.52. Supporting the present study, Emadian et al. (2021) results coincided and showed that the frictional resistance was 1.59 [9] which was more than the control group. On the contrary, Hosseinzadeh et al. (2013) showed that CHG with 0.412 of frictional resistance had no statistically significant difference from its control group. Also, the contact period (immersion for 1.5 hours) of CHG with bracket and wire surfaces was important which didn’t exactly stimulate the clinical situation [21]. Supporting the current results, a study done by Alwafe et al. (2019) NaF mouthwash demonstrated a high mean friction resistance of 0.92 and the least for the AS with 0.66 [22]. The findings of this investigation were consistent with those of other studies where the frictional coefficient for NAF was 1.60. On the contrary, Kao et al. (2006) showed that the SS wires had the lowest frictional resistance in the 0.2% acidulated flurophasphate condition, with a lower value of 1.40 compared to AS (1.63) [23]. In the current study, the coefficient of friction for the CC mouthwash with SS archwire and bracket interface was 1.45.

No literature was found regarding frictional characteristics and which measured the coefficient of friction between the orthodontic SS brackets to SS archwires with charcoal products. The current study revealed a coefficient of frictional resistance of 1.157 for O_3_. No confounding articles were found regarding the frictional resistance with SS brackets and the archwires. When comparing the dry condition to the other groups, it was evident that all the substances significantly affect the coefficient of friction. AS, CHG, CC, NaF, and O_3_ all exhibit statistically significant differences in friction compared to dry conditions. These findings suggest that the presence of these substances could either increase or decrease the coefficient of friction, indicating a significant influence on the lubricating or adhesive properties of surfaces.

The comparisons between the different substances also provide valuable insights. AS shows significant differences in friction compared to both CHG and CC types of mouthwash. This suggests that AS may have distinct lubricating properties compared to these substances. Similarly, the comparison between AS and O_3_ reveals a significant difference in friction, implying that O_3_, known for its potent antimicrobial and lubricating properties, could significantly alter the coefficient of friction when compared to AS. On the other hand, some comparisons did not yield statistically significant differences. For instance, there were no discernible variations in friction between AS and NaF. This suggests that these two substances may have similar effects on the coefficient of friction within the tested context.

Limitations

Experimental setup and research setting may not exactly mimic the clinical conditions. Post-evaluation of physical characteristics and surface topography of the archwires was not performed.

Further scope

Further studies are needed to fully understand the mechanisms underlying the observed effects and to evaluate the practical implications in clinical or oral care settings. The acceptability of the various types of mouthwashes and their frequency and intensity needs further investigation.

## Conclusions

Ozone-infused oil-pulling solution with coconut oil showed the least coefficient of friction, reducing the frictional resistance among the stainless-steel brackets and archwires. Significant differences in coefficient of friction were observed between the bracket-archwire interface, at dry conditions (control group) to all other test groups, especially with ozone-infused oil-pulling solution with coconut oil. No significant differences in coefficient of friction were observed in the bracket-archwire interface between chlorhexidine gluconate mouthwash with charcoal mouthwash, as well as between artificial saliva and sodium fluoride mouthwash.
